# Assessing cognitive function in longitudinal studies of ageing worldwide: some practical considerations

**DOI:** 10.1093/ageing/afad122

**Published:** 2023-10-30

**Authors:** Céline De Looze, Joanne Feeney, Katrin M Seeher, Jotheeswaran Amuthavalli Thiyagarajan, Theresa Diaz, Rose Anne Kenny

**Affiliations:** The Irish Longitudinal Study on Ageing, School of Medicine, Trinity College Dublin, Dublin 2, Ireland; The Irish Longitudinal Study on Ageing, School of Medicine, Trinity College Dublin, Dublin 2, Ireland; Brain Health Unit, Department of Mental Health and Substance Use, World Health Organization, Geneva, Switzerland; Ageing and Health Unit, Department of Maternal, Newborn, Child, Adolescent Health and Ageing, World Health Organization, Geneva, Switzerland; Epidemiology, Monitoring and Evaluation Unit, Department of Maternal, Newborn, Child, Adolescent Health and Ageing, World Health Organization, Geneva, Switzerland; The Irish Longitudinal Study on Ageing, School of Medicine, Trinity College Dublin, Dublin 2, Ireland; Mercer’s Institute for Successful Ageing, St. James’s Hospital Dublin, Dublin 8, Ireland

**Keywords:** ageing, cognitive capacity, longitudinal studies, early detection, cross-countries comparison, older people

## Abstract

Over 55 million people live with dementia worldwide. With 40% of modifiable risk factors estimated to contribute to dementia, the potential for prevention is high, and preventive measures, at an early stage of cognitive decline, are likely to positively influence future dementia trends. Countries need reliable health data and adequate measurement tools to quantify, monitor and track early changes in cognitive capacity in the general population. Many cognitive tests exist; however, there is no consensus to date about which instruments should be employed, and important variations in measurement have been observed. In this narrative review, we present a number of cognitive tests that have been used in nationally representative population-based longitudinal studies of ageing. Longitudinal panel studies of ageing represent critical platforms towards capturing the process of cognitive ageing and understanding associated risk and protective factors. We highlight optimal measures for use at a population level and for cross-country comparisons, taking into consideration instrument reliability, validity, duration, ease of administration, costs, literacy and numeracy requirements, adaptability to sensory and fine motor impairments and portability to different cultural and linguistic milieux. Drawing upon the strengths and limitations of each of these tests, and the experience gained and lessons learnt from conducting a nationally representative study of ageing, we indicate a comprehensive battery of tests for the assessment of cognitive capacity, designed to facilitate its standardised operationalisation worldwide.

## Key Points

- Reliable measurement tools are needed to quantify and track early changes in cognitive capacity in the general population.
- Many cognitive tests exist; however, there is no consensus to date about which instruments should be employed.
- We compare a number of cognitive tests, which have been used in longitudinal studies of ageing.
- We indicate a comprehensive battery of tests designed to facilitate a standardised assessment of cognitive capacity worldwide.

## Introduction

Over 55 million people live with dementia worldwide. With 40% of modifiable risk factors estimated to contribute to dementia, the potential for prevention is high, and preventive measures, at an early stage of cognitive decline, are likely to positively influence future dementia trends. Countries need reliable health data and adequate measurement tools to quantify, monitor and track early changes in cognitive capacity in the general population. Many cognitive tests exist; however, there is no consensus to date about which instruments should be employed. In this paper, we review a number of cognitive tests that have been used in nationally representative population-based longitudinal studies of ageing. Based on the experience gained and lessons learnt from conducting a nationally representative study of ageing, we highlight practical aspects to consider and indicate a comprehensive battery of tests for the assessment of cognitive capacity worldwide.

### Cognitive capacity in ageing

The operationalization of health in the ageing context has undergone an important paradigm shift in recent decades, moving away from a focus on diseases and disease severity to a more holistic model that emphasises the functional capacity of an individual. The World Health Organization (WHO) World Report on Ageing and Health (2015) [1] defines healthy ageing as ‘the process of developing and maintaining the functional ability that enables well-being in older age’. At the core of this approach is the concept of intrinsic capacity, which can be defined as a composite of the physical, mental and psychosocial capacities (as opposed to deficits) that an individual can bring to bear at any point in time [2]. Intrinsic capacity is conceptualized upon five domains—cognitive capacity, locomotion, sensory, psychology and vitality [3]. As there is no standard definition of cognitive capacity as it is conceptualized in the WHO report, cognitive capacity refers herein to an individual’s mental capacity and spans a large spectrum of cognitive domains, encompassing memory, attention, executive function, perception, visuo-construction, orientation and language abilities.

### Longitudinal studies of ageing: a tool to monitor cognitive capacity changes in the general population

Assessing and monitoring cognitive capacity in older adults requires the use of appropriate measurement tools. In this narrative review, we present a number of cognitive tests that have been used in nationally representative population-based longitudinal studies of ageing to assess cognitive function in older adults (see Table 1 for an overview). Longitudinal panel studies of ageing present an ideal pathway to monitor dynamic changes in cognitive function at a population level as they obtain repeated measures from representative samples. Broad by nature, they collect rich data pertinent to cognitive ageing that can be leveraged to answer important research questions pertaining to brain health worldwide. Being population-based, they employ tests that have been chosen for their ecological validity at the population level. We focus on the cognitive tests that have been employed in the Health and Retirement Study (HRS) international family of studies, a growing network of longitudinal studies of ageing modelled on the University of Michigan HRS in the USA. HRS is a longitudinal population-based study of 20,000 adults aged 51+ and has been collecting a vast array of health, economic and social data since 1992 [4]. Many other countries, including low-income and middle-income countries, have followed suit, developing longitudinal population-based studies using harmonized survey questions and methods. We also include the cognitive tests employed in the Harmonized Cognitive Assessment Protocol (HCAP) [5] international research network, which was developed by the HRS network to collect rich, harmonized measures of cognitive function across HRS studies as well as the tests employed in the Aging, Demographics and Memory Study (ADAMS) [6] and the 10/66 dementia studies [7] that served as the starting point for developing HCAP (see Appendix S1 for further description).

**Table 1:** Overview of the cognitive tests administered, and the cognitive domains measured across HRS core and HCAP studies

Cognitive tests	10/66	ADAMS	CHARLS	CRELES	ELSA	ELSI	HAALSI	HAGIS	HART	HRS	IFLS	JSTAR	KLoSA	LASI	MARS	MHAS	NICOLA	SAGE	SHARE	TILDA
Global cognitive function																				
MMSE	x	x	x				x						x	HMSE		x	x			x
MOCA																	x			x
TICS		x	x		x		x				x			x		x				x
CSI-D 4	x	x	x		x		x							x		x				x
Memory																				
Self-rated memory		x	x		x	x	x	x	x	x	x				x	x	x	x	x	x
Word Recall– Immediate	x	x	x	x	x	x	x	x		x	x	x	x	x	x	x	x	x	x	x
Word Recall– Delayed	x	x	x	x	x	x	x	x		x	x	x	x	x	x	x	x	x	x	x
Word Recall – Recognition		x	x		x		x							x		x				x
Logical Memory – Immediate		x	x		x		x							x		x				x
Logical Memory – Delayed		x	x		x		x							x		x				x
Logical Memory –recognition		x			x		x							x						x
Prospective Memory					x	x				x							x			x
Attention/working memory/executive function																				
Letter/symbol cancellation			x		x		x			x				x		x	x		x	x
Symbol Digit		x	x		x		x	x								x				x
Digit Span		x	x	x										x				x		
Color Trail Test/Trail Making Test		x	x		x												x			x
Three-stage Command				x									x	x			x			x
Raven’s matrices					x		x	x						x						x
Numeracy/numeric ability																				
Serial 7 s			x	x	x				x	x	x	x	x	x	x	x	x		x	x
Backward counting					x					x	x			x	x	x				x
Number series			x		x			x		x	x			x						x
Computation questions					x					x		x		x					x	x
Language/fluency																				
Verbal Fluency	x	x	x		x	x	x	x		x	x			x	x	x	x	x	x	x
Object naming		x	x		x	x				x			x	x	x		x			x
Vocabulary								x		x										x
Word spelling																	x			x
Repeat sentences/phrases													x				x			x
Write sentences			x										x	x			x			x
Reading comprehension													x	x			x			x
Orientation																				
Date			x	x	x	x		x	x	x	x	x	x	x	x	x	x		x	x
Day of the week			x	x	x	x	x	x		x		x	x	x	x		x		x	x
Season													x				x			x
Person					x	x				x					x					
Place												x	x	x			x			x
Visuo-construction skills																				
Constructional praxis – immediate		x	x		x		x				x		x	x		x	x			x
Constructional praxis – delayed		x			x		x							x		x				x

### Cognitive testing instruments for the assessment of cognitive capacity

#### Some considerations

A number of cognitive tests have been used in the HRS and HCAP studies (hereafter termed ‘HRS/HCAP’) to measure global cognitive function and domain-specific cognitive function. Together, these tests can provide a rich picture of an individual’s cognitive capacity; however, many other factors influence test performance including test administration and educational, cultural, social, linguistic and ethnic differences. Thorough considerations should be given to instrument reliability, validity, duration, ease of administration, costs, literacy, numeracy, sensory and motor requirements and portability to different cultural and linguistic milieux. These latter practical considerations reflect our experience and lessons learnt from conducting a population-based study of ageing, extensive reviews of other HRS and HCAP study protocols, notably those from surveys conducted in low- and middle-income countries, as well as frequent discussions on cognitive testing within the HRS network.

The degree to which an instrument adequately reflects the cognitive construct it claims to measure, the internal consistency or homogeneity of the items it is composed of and its sensitivity and specificity to correctly identify differences and changes in cognitive function are important psychometric properties to consider [8, 9]. Other important psychometric features include inter-rater and intra-rater reliability [8] as well as susceptibility to learning effects that may occur when an individual is repeatedly exposed to the same test and that may increase an individual’s performance as they become familiar [10].

Brevity and ease of administration are other important elements in population settings. Limiting the number of tests and their length is key towards reducing participant burden and fatigue. Simplicity of the administration and scoring protocol also facilitates the standardised administration across personnel and studies, ensuring data quality. The need and time taken for quality control should also be assessed. The costs involved in administering cognitive tests, including test licences, equipment and personnel training, are additional aspects to take into account, particularly to assess feasibility of use in low- and middle-income countries.

Worldwide implementation and comparison across countries also poses challenges with respect to literacy and numeracy and more generally to formal educational attainment. While literacy rates have been steadily increasing globally, there remains a considerable disparity between high- and low–middle-income countries, most notably among older adults [11]. In countries where illiteracy and innumeracy are high, care should be taken to assess the impact that these may have on test performance [12]. Efforts should be made to include cognitive tests that are not sensitive to educational attainment, literacy or numeracy, in order to help disentangle true cognitive differences from education effects.

There are also stark differences between high- and middle- or low-income countries in the prevalence of visual and hearing impairment among older adults [13, 14]. Poor hearing and vision can affect performance on cognitive testing [15] and thereby bias estimations of cognitive capacity and prevalence of cognitive impairment across countries. These considerations are also important for minimising the risk of selection bias into studies [16].

Finally, assessing the cross-country portability of a cognitive test is essential to determine whether differences in cognitive function between countries reflect genuine differences in risk and prevalence or whether they result from poor translation or lack of a cultural/linguistic equivalent [17]. Cognitive tests generally rely on Western cultural concepts, and many were originally developed in English. Modifications reflecting adaptation to different cultural milieux and languages may be considered prior to implementation to ensure instrument validity and reliability [16], including use of alternative concepts and vocabulary and translations to other languages and/or dialects [16, 18].

We discuss, hereafter and per cognitive domain, the advantages and disadvantages of the cognitive tests used in the HRS and HCAP studies, in light of the above considerations. Table 2 provides descriptions of the cognitive tests themselves. Appendix S2 provides for each of these tests more detailed information on validity, reliability, validity, duration, ease of administration, costs, literacy and numeracy requirements, adaptability to sensory and fine motor impairments and portability to different cultural contexts.

**Table 2:** Brief descriptions of the cognitive tests administered and cognitive domains measured in the HRS and HCAP studies. The reader should refer to the references listed in the table for more detailed descriptions of the cognitive tests and domains

Cognitive domain	Cognitive tests	Test overview
Global cognitive function	MMSE (Mini-Mental State Examination) [20]	Assesses an individual’s global cognitive abilities; it is composed of 12 tests subsumed under six different cognitive domains including orientation (time, date, place), registration (3-word list immediate recall), attention and calculation (Serial 7 s; WORLD backward spelling), verbal memory (3-word list delayed recall), language (naming, repetition, 3 stage command, written command, sentence writing) and visuo-constructional skills (figure copy).
	MoCA (Montreal Cognitive Assessment) [21]	Assesses an individual’s global cognitive abilities; it is composed of 14 tests subsumed under six different cognitive domains including visuo-constructional/executive function skills (figure copy, clock drawing and trail test), verbal memory (word delayed recall), attention/working memory (sustained attention, serial 7 s, forward and backward digit span), language/executive function (naming, sentence repetition and phonemic fluency), conceptual thinking (verbal abstraction) and orientation (date and place).
	Telephone Interview for Cognitive Status (TICS) [22]	Assesses an individual’s global cognitive abilities; it is composed of 22 questions related to orientation (date, season, place), numeracy (backward counting from 20), verbal memory (10-word list immediate and delayed recalls), attention and calculation (Serial 7 s), naming (objects, president and vice-president) and language (sentence repetition, abstract thinking). The HCAP battery only includes three of these questions that assess orientation and vocabulary.
	Community Screening Instrument for Dementia [23]	Assesses an individual’s global cognitive abilities; it consists of two components: a cognitive assessment and an informant interview. The cognitive tests (32 items in total) assess verbal fluency (animal naming), language (naming, sentence repetition and comprehension), verbal memory (story immediate recall), attention/calculation, orientation (date, place, mayor), attention (ability to follow instructions) and visuo-construction skills (drawing circles and pentagons). In the HCAP battery, a brief CIS-D (four items) used in 10/66 is used and evaluates language, knowledge and attention.
Memory	Self-rated memory	Assesses an individual’s own perception of their memory. The respondent may be asked to rate their memory on a scale from excellent to poor.
	CERAD Word Recall [32] – Immediate	Assesses an individual’s episodic/verbal memory or ability to recall 10 orally presented words. The respondent is presented a list of words and is asked to recall them immediately after being heard. Typically, three trials are administered and a total immediate recall score (out of 30) is computed.
	CERAD Word Recall – Delayed	Assesses an individual’s episodic/verbal memory or ability to recall orally presented words. The respondent is asked to recall the same list of words after a time delay (e.g. 15 minutes after they heard the list of words for the first time).
	CERAD Word Recall –Recognition	Follows the delayed recall test and assesses an individual’s ability to correctly identify words previously presented as part of the CERAD word recall task. The respondent is shown 10 previously seen words along with 10 foils (i.e. not part of the previously presented list) and asked whether each word, in turn, was part of the list.
	HRS 10-Word Recall [34] – Immediate	Assesses episodic verbal memory and is very similar to the CERAD word recall test. Respondents are shown a list of 10 words (from four possible lists) and asked to recall them immediately. The number of immediate recall trials varies from 2 to 3 depending on the study.
	HRS 10-Word Recall – Delayed	Respondents are asked to recall the same 10 words after a delay (e.g. 15 minutes after first presentation).
	Wechsler Logical Memory Test [40] – Immediate	Assesses episodic memory. Respondents hear a story ‘Anna Thompson’ with 25 story points. Immediately following, they are asked to recall everything they can remember about the story.
	Wechsler Logical Memory – Delayed	Assesses episodic memory. After a delay, respondents are asked to recall any story points from the LMT.
	Wechsler Logical Memory – Recognition	This follows the delayed recall test. Sixteen story items are read aloud to the respondent, eight true (i.e. were part of the original story) and eight false items. Respondents must indicate whether or not they believe the item was in the story.
	East Boston Memory Test [41] – Immediate	Assesses episodic memory. Also known as the ‘Brave Man’ story test. Very similar to the LMT but shorter (six story points). Respondents hear a short story read aloud and are immediately asked to recall as much of the story as possible.
	East Boston Memory Test – Delayed	After a delay, respondents are asked to recall any story points from the ‘Brave Man’ story.
	Prospective memory	Assesses an individual’s ability to remember to perform an action in the future, often in response to a particular cue. Assessed via two tasks: (i) respondents are given instruction to write their initials in the top left-hand corner of a page attached to a clipboard when it is handed to them. (ii) Respondents are instructed to remind the interviewer to record the time when the interviewer announced that the cognitive section has finished. The first task is closely based on a task incorporated in the MRC Cognitive Function and Ageing Study (MRC CFAS) [45], and the second is a similar task to one used in the Rivermead Behavioral Memory Test [46]. HRS core study includes a prospective memory task in two waves. This task involves asking respondents to remind the interview to check the computer in 5 minutes.
Attention/working memory/ executive function	Letter cancellation [49]	Assesses visual–spatial scanning, sustained and selective attention, psychomotor speed and motor coordination. For example, the respondent may be asked to cross out as many Ps and Ws of a list of letters as possible within a defined timeframe.
	Symbol cancellation [48]	Assesses attention and psychomotor speed. Respondents are given a page full of symbols and shown a target symbol that is present among the different symbols on the sheet. They must circle the target symbol, working as quickly as possible. Correctly circled symbols and errors are noted.
	Symbol Digit Modalities Test (SDMT) [52]	Assesses processing speed (cognitive and motor), visual scanning and working memory. It is paper–pencil measure that requires respondents to substitute numbers for abstract symbols using a reference key provided.
	Digit span [50]	Assesses attention and verbal working memory. Respondents are asked to repeat sequences of numbers forwards and backwards. The highest number of digits that a respondent can repeat successfully in the forward or backward order is their digit span in that condition. An abbreviated test is included in the MMSE.
	Color Trails Test (CTT1&2) [53]	Assesses visual scanning/processing speed and executive function (including working memory and sustained/divided attention); the respondent is asked to use a pencil to rapidly connect circles numbered in consecutive order (e.g. from 1 to 25) in CTT1. In CTT2, the respondent is asked to once again rapidly connect circles in consecutive order but this time alternating colours.
	Trail Making Test (TMT A&B) [54]	Assesses visual scanning/processing speed and executive function (including working memory and sustained/divided attention); the respondent is asked to use a pencil to rapidly connect circles numbered in consecutive order (e.g. from 1 to 25) in TMTA. In. TMTB, the respondent must once again connect circles in sequence but this time alternating between the number and letters of the alphabet in consecutive order (e.g. 1-A-2-B-3-C).
	Three-stage command task [20]	Assesses executive function, requiring three actions in sequence. Most commonly, the respondent is asked to take a paper in their right hand, fold it in half and put it on the floor. The three-stage command forms part of the MMSE in studies that include the latter, though it can also be used as a stand-alone item (e.g. CRELES).
	Raven’s matrices [51]	Ravens’s Standard Progressive Matrices assesses abstract reasoning and is a measure of fluid intelligence. A 17-item form is used in HRS/HCAP. For each item, the respondent is shown a black-and-white pattern with a piece missing and asked to select the missing piece out of six possible options.
Numeracy/ numeric ability	Serial 7 subtraction [20, 70]	Assesses numeracy, concentration and working memory (or the ability to hold and manage information temporarily). Serial 7 s is a descending subtraction task that assesses numeracy, concentration, processing speed and working memory. It is a component of the MMSE and MoCA. In this test, the respondent is asked to subtract 7 to 100 for a sequence of five subtractions.
	Backward counting	Assesses executive function and the ability to apply simple numerical concepts; the respondent may be asked to count backward from 20 to 0, or they may be asked to count backward from 100 as fast as possible within 30 seconds.
	Number series [71]	The number series test assesses quantitative reasoning or fluid intelligence. It involves reasoning with concepts that depend upon mathematical relationships. For example, the respondent may be asked to look at a series of numbers with a number missing from the series and determine the missing number in the series given the numerical pattern (e.g. 2 4 6 MISSING 10).
	Computation/Logic	Assesses numeracy across different types of mathematical skills (subtraction, division, fraction, percentage). For example, the respondent may be asked, ‘If the chance of getting a disease is 10 in 1,000, what percent of people will get the disease?’.
Language/fluency	Verbal fluency [76, 77]	This task draws on word knowledge, semantic memory and executive processes such as set shifting and inhibition (or the ability to suppress inappropriate/incorrect items). The respondent is asked to generate as many words as possible, either from a given semantic category (e.g. animals; also called ‘semantic fluency’) or beginning with a particular letter (e.g. ‘F’; also called ‘phonemic fluency’), within a short time period (e.g. in 1 minute).
	Object naming [22]	Two objects are described to respondents (most often ‘cactus’ and ‘scissors’), and they must name them. Respondents are also asked the name of the current president/prime minister.
	Vocabulary	Assesses established knowledge or crystallized intelligence. Assessed in HRS/HCAP with the word pronunciation test (NART) [78], a vocabulary test adapted from the WAIS-R [79] or a multiple choice test devised for the COGNITO study [80]. These tests can be valuable indicators of prior cognitive ability, as they are typically age invariant and stable into the early stages of dementia and other forms of cognitive impairment [89].
	Repeat sentences/phrases [20]	Assesses the ability to repeat phrases or sentences exactly as said.
	Word spelling [20]	Respondents are asked to spell the word ‘world’ forwards and then backwards.
	Write sentences [20]	Assesses writing skills.
	Reading comprehension (Read and follow instructions) [20]	Assesses the ability to read and follow instructions. For example, the respondent may be asked to read on a piece of paper ‘close your eyes’ and to act it out.
Orientation [20]	Day, Month, Year, Day of the week Season Person Place	Assesses an individual’s awareness of time, their position in places and knowledge of famous persons. For example, the respondent may be asked what day it is or who the prime minister is. These items are components of the MMSE and MoCA and CSID but may also function as a stand-alone set of questions.
Visuo-construction [20, 21, 32, 33]	Constructional praxis and constructional praxis recall	Picture drawing: assesses an individual’s ability to integrate and coordinate visual and fine motor skills, it measures their ability to manipulate spatial information to make a design. The respondent is asked to copy a series of two- and three-dimensional shapes. Constructional praxis items can also be found as part of the MMSE and MoCA tests. Recall: usually followed by a recall task that assesses an individual’s visual memory and visuospatial constructional abilities; for example, the respondent may be asked to recall a figure that they previously copied during the interview.

#### Global cognitive function

Global cognitive tests are often used as screening tools for cognitive impairment or dementia, have the advantage of giving a snapshot of performance across multiple cognitive domains and are quick to administer, albeit lacking depth in any one domain [19]. HRS/HCAP have used the Mini Mental State Examination (MMSE) [20], the Montreal Cognitive Assessment (MoCA) [21], the Telephone Interview for Cognitive Status (TICS) [22] and the brief Community Screening Instrument for Dementia (CSI-D) [23]. The MMSE and MoCA have shown relatively good validity and reliability for the assessment of dementia and mild cognitive impairment, respectively, in clinical settings [20, 21, 24], the purpose for which they were developed. The MMSE and MoCA original versions also demonstrate adequate-to-excellent internal consistency and construct validity, adequate-to-excellent test–retest reliability (although susceptible to learning effects [25, 26]) and adequate-to-excellent inter-rater and intra-rater reliability in community-dwelling older adults [24, 27, 28]. The TICS also shows a high level of internal consistency, test–retest and inter-rater reliability in older adults [29], with vulnerability to learning effects too [30].

The MMSE, MOCA, TICS and CSI-D are accessible online, with associated costs for the MMSE and TICS. They are all quick and easy to administer, although the scoring of specific items can be prone to error. The MMSE and MOCA are susceptible to ceiling effects in healthy cohorts. They tend to be biased against poor education and visual impairment; however, modified versions are available to address these limitations. The TICS can be used to examine individuals with low education or visual impairment, while the CSI-D does not require literacy. The MMSE, MoCA and TICS have been validated in many countries and translated into many languages; the CSI-D has been extensively validated across a variety of low- and middle-income countries. All tests necessitate modifications for use in different cultural and linguistic milieux.

#### Memory

The encoding, recall and recognition of previously learned verbal information (e.g. word lists, word pairs and events/stories) undergoes notable decline with age [31]. As such, verbal memory assessment forms a core component of the cognitive batteries in the main HRS studies as well as in the HCAP studies. The verbal memory tasks included in HRS/HCAP are the Consortium to Establish a Registry for Alzheimer’s Disease (CERAD) word-list memory test [32, 33] and the HRS 10-word list recall test [34]. Both tests are relatively easy to administer and score. Both the CERAD word-list memory test (if verbally administered) and the HRS 10-word list recall tests are appropriate for populations with low literacy. Poor hearing on the part of the respondent may pose a challenge as the words cannot be repeated. The CERAD task has already been translated and validated in a number of languages [18]. CERAD word list learning and recall, particularly the delayed recall task, has been shown to have good sensitivity and specificity for mild cognitive impairment and dementia [33, 35–37], with high inter-rater and test–retest reliability [33, 38]. The HRS word recall immediate and delayed recall tasks have shown good convergent and divergent validity [39]; however, information on the other measurement properties of the HRS recall tasks is lacking.

Story memory is assessed using the Wechsler Logical Memory test (LMT) [40] and the East Boston Memory test (EBMT) [41]. The LMT exhibits good psychometric properties including test–retest reliability [42, 43], with the magnitude of practice effects varying as a function of age [44]. While administration is straightforward, accurate scoring is difficult without audio recording the respondent. Nonetheless, the EBMT has exhibited reasonable sensitivity to dementia, correctly classifying 78% of outpatients in a memory clinic setting [41]. Both story memory tests can be adapted for different cultural contexts and do not require literacy. Poor hearing on the part of the respondent may pose a challenge.

Prospective memory (i.e. memory for an intended action) [45, 46] is also assessed in several of the HRS studies via two tasks. There is little performance variability, however, given that there are only two outcomes, which limits the sensitivity of these tasks to changes in functioning. Furthermore, to the best of our knowledge, data on the psychometric properties of the tasks have not been published.

Respondents are also asked to rate their own memory function in the core HRS studies. This does not require literacy or formal education and can still be used in cases of visual impairment or presented visually for individuals with hearing loss. This task may, however, be sensitive to cultural differences.

#### Attention/working memory/executive functioning

There are a number of tests in HRS/HCAP that assess attention/processing speed and executive function. Speeded tasks generally show the earliest age-related performance declines, and speed of processing markedly affects performance across most domains [47]. Tests include the Double Letter Cancellation Test/Symbol Cancellation Test [48], which assesses attention and processing speed [49]. The Digit Span [50], Raven’s Progressive Matrices (RPM) [51] and Three Stage Command [20] assess working memory and executive functioning. The Symbol Digit Modalities Test (SDMT) [52], Color Trails Test (CTT; Part 1 & 2) [53] and Trail Making Test (Part A & B) [54] assess both attention/speed and executive functioning.

The Double Letter Cancellation task does not require a licence and is quick and relatively easy to administer. It is not culturally neutral; however, as respondents need to recognise letters of the alphabet (it has been translated into several different languages). Construct validity has been demonstrated for this task [55]. The Symbol Cancellation Task is a good alternative to letter cancellation for populations with low literacy.

The SDMT is relatively quick to administer and score but with an associated cost for materials. It has good construct validity, loading on processing speed factors with some degree of learning involved [56], as well as good test–retest reliability [52], though it may be influenced by practice effects if alternate test forms are not used [57]. It does not require literacy but is affected by numeracy and educational attainment [58]. It is not appropriate for individuals with visual impairment.

There is no cost associated with the Digit Span test and it is quick and easy to administer and score. The task is administered verbally and so may be challenging for individuals with very poor hearing. It is appropriate for international comparisons, though will be influenced by education [59].

The Trail Making Test (TMT) has demonstrated adequate construct validity [60, 61] and test–retest reliability [54]. No practice effects were found over larger time intervals (e.g. 1 year) [62]; however, it is susceptible to practice effects at short time intervals [63] (alternate forms exist for this purpose [64]). It also has excellent inter-rater reliability [65]. The CTT also has acceptable psychometric properties [53, 66]. One advantage of the TMT over the CTT for inclusion in a test battery is that it does not require a licence to administer. There is some cost associated with both tests. Both are demanding to administer and score and require detailed training and close attention on the part of the administrator during the testing process. Both tests require ability to hold a pen and draw on paper and therefore are not appropriate for use in individuals with fine motor disability. While these tests require good visual acuity, the TMT has the additional requirement of literacy whereas the CTT does not use letters and therefore maintains cultural equivalence.

The three-stage command test is largely the same across studies with minor variation to accommodate individuals with certain physical disabilities. No literacy requirements or specific test materials are required (a blank piece of paper only), and it is applicable across cultures.

The RPM has demonstrated adequate construct validity [67, 68] and adequate-to- excellent test–retest reliability [51, 67]. The 17-item version used by many of the HRS-HCAP studies is not long and is culturally fair with no literacy requirement. This version is also quick and easy to administer and score. However, data on the psychometric properties of the 17-item version are lacking. There is a licence needed for administration. It also requires good functional visual acuity making it inappropriate for populations with severe visual impairment.

#### Numeracy/numeric ability

Numeric ability has important real-world applications for older adults, including financial decision-making, weighing up healthcare choices and maintaining general independence [69]. To assess numeric ability, HRS/HCAP have employed the Serial 7 s subtraction task [20, 70], backward counting, number series [71] and other computation questions.

Adequate construct and discriminant validity of the Serial 7 s as measures of concentration and processing speed have been reported [72]; however, it is heavily influenced by basic arithmetic skills [73]. The administration of Serial 7 s and backward counting is simple and quick. These tests do not require any specific equipment, except a stopwatch for backward counting. The scoring of both tasks is somewhat challenging, benefitting from quality control to ensure scoring reliability.

Number series has shown solid construct and discriminant validity for quantitative reasoning ability among community-dwelling older adults [71]. Computation questions assess numeracy across different types of mathematical skills and encompass, e.g. subtraction, division, fraction and percentage. Number series and computation questions are relatively quick to administer and score, and they do not require any specific equipment.

As numeracy tests, Serial 7 s, backward counting, number series and computation questions may be difficult for individuals with low education. Administered orally, they are useful for examining individuals with visual impairments or fine motor impairments. They can also be directly implemented in different cultural milieux and easy to use for cross-country comparisons.

#### Language/fluency

Language and language fluency are important facets of human cognition that evidence divergent trajectories with age. For example, while vocabulary remains stable or even improves with age, verbal fluency is sensitive to age-related decline and different types of neuropathology [74, 75].

Language and fluency are assessed in HRS/HCAP using verbal fluency [76, 77], object naming [22], vocabulary [78–80], backward word spelling [20], sentence repetition [20], sentence writing [20] and reading/comprehension [20]. The latter three tasks form part of the MMSE scale.

Animal naming is a widely used semantic fluency task. It is free to use and requires no specific testing materials other than a timer. Furthermore, it does not require literacy and is easily transferable to different cultures/languages [18]. Phonemic fluency is assessed as part of the MoCA. Both fluency tasks are valid measures of verbal ability and executive functioning [81] and demonstrate adequate test–retest reliability [82]. Both are appropriate for individuals with visual or hearing impairment, though not with speech difficulties.

Object naming is assessed via several questions included in the TICS and MMSE. These tests are quick and easy to administer and score. Literacy is not a requirement, and these tests are also suitable for individuals with visual impairment. Tasks are transferable across countries with substitutions of items to retain cultural relevance.

Vocabulary is assessed in a subset of HRS/HCAP studies using the National Adult Reading Test (NART) [78], a vocabulary test adapted from the Wechsler Adult Intelligence Scale - Revised (WAIS-R) [79] and a multiple choice test adapted from the COGNITO study [80]. The validity of the NART as a measure of prior intellectual functioning is established [83, 84]. The NART requires high levels of literacy and is therefore sensitive to cultural and education differences. It can also be difficult to score as regional accents influence pronunciation and is only currently available in English. The vocabulary test adapted from the WAIS-R is preferable in this as it does not require literacy and exhibits excellent psychometric properties [85]. As the WAIS-R and COGNITO vocabulary test rely on giving word definitions rather than on pronunciation of words, they are theoretically more comparable across countries/languages.

Backward word spelling, sentence repetition, sentence writing and reading/comprehension are all quick and easy to administer individual components of the MMSE. Backward word spelling shows good construct validity [72] though poses some challenges in scoring. Word spelling and sentence writing require respondents to be literate and sentence writing is also not appropriate for individuals with poor vision. These items are not valid in diverse cultural settings [18].

#### Orientation

Orientation tests are quick and easy to administer and score. They do not require literacy or numeracy skills and can be administered to individuals with sensory or fine motor impairments. Adaptations to the cultural milieu may be needed for some orientation items. While they are rapidly able to distinguish the most severely cognitively impaired individuals, at the population level these items often suffer from ceiling effects.

**Table 3:** Strengths (√) and limitations (X) of the cognitive tests used in the HRS and HCAP studies for use at a population level and across countries

Cognitive domain	Cognitive tests	Costs	Timing (<10 minutes)	Ease of administration	Ease of scoring	Validity/ reliability	Feasibility of use for individuals with low literacy/numeracy	Feasibility of use for individuals with sensory or physical impairments			Linguistic/ cross-cultural portability
								Poor vision	Poor hearing	Fine motor/ speech impairment	
Global cognitive function	Mini-Mental State Examination (MMSE)	X	√	√	√	√	X ^a^	X	X	X	X ^a^
	Montreal Cognitive Assessment (MoCA)	√	√	√	√	√	X ^a^	X	X	X	X ^a^
	Telephone Interview for Cognitive Status (TICS)	X	√	√	√	√	√	√	X	√	X
	Community Screening Status Interview for dementia (CSI-D) (Brief CSI-D 4 items)	√	√	√	√	√	√	√	√	√	√
Memory	Self-rated memory	√	√	√	√	N/A	√	√	√	√	√
	Word Recall –Immediate	X ^b^	√	√	√	√	√	√ ^c^	X	√	X ^a^
	Word Recall –Delayed	X	√	√	√	√	√	√	X	√	X ^a^
	Word Recall –Recognition	X	√	√	√	√	√	√	√	√	X ^a^
	Logical Memory – Immediate	X	√	√	X	√	√	√	X	√	X ^a^
	Logical Memory – Delayed	X	√	√	X	√	√	√	√	√	X ^a^
	Logical Memory– Recognition	X	√	√	X	√	√	√	√	√	X ^a^
	Prospective memory	√	√	√	√	N/A	√	√	√	√	X
Attention/working memory/executive function	Letter cancellation	X	√	√	X	√	X	X	√	X	X
	Symbol digit	X	√	√	√	√	√	X	√	√	X ^a^
	Digit span	√	√	√	√	N/A	X	√	√	√	X
	Trail Making Test	X	X	X	X	√	√	X	√	X	X
	Color Trail test (1&2)	X	X	X	X	√	X	X	√	X	√
	Three-stage command task	√	√	√	√	N/A	√	√	√	√	√
	Raven’s Matrices (17-item)	X	√	√	√	√	√	X	√	√	√
Numeracy/numeric ability	Serial 7 subtraction	√ ^d^	√	√	X	√	X	√	√	√	√
	Backward counting	√	√	√	X	N/A	X	√	√	√	√
	Number series	√	X	√	√	√	X	√	√	√	√
	Computation/Logic	√	√	√	√	N/A	X	√	√	√	X
Language/fluency	Verbal fluency	√	√	√	√	√	X	√	√	X	X
	Object naming	√	√	√	√	N/A	√	√	√	√	X ^a^
	Vocabulary	√	√	√	X	√	X	√	√	√	X
	Word spelling	√ ^d^	√	√	X	√	X	√	√	√	X
	Repeat sentences/phrases	√ ^d^	√	√	√	N/A	√	√	X	X	X ^a^
	Write sentences	√ ^d^	√	√	√	N/A	X	X	√	X	X
	Reading comprehension	√ ^d^	√	√	√	N/A	X	X	√	√	X
Orientation	Day, Month, Year, Day of the week	√ ^d^	√	√	√	N/A	√	√	√	√	√
	Season										√
	Person										X ^a^
	Place										X ^a^
Visuo-construction	Constructional praxis	X	√	√	√ ^e^	√	√	X	√	X	√
	Constructional praxis recall – delayed	X	√	√	√ ^e^	√	√	X	√	X	√

N/A, information not available to our knowledge.

^a^The test is not transferable as is; however, modified versions exist.

^b^Costs applicable to the CERAD 10-word recall only.

^c^The CERAD 10-word recall can be administered orally in the presence of poor vision.

^d^MMSE costs not applicable if the item is used as a stand-alone test.

^e^While the circle, diamond and overlapped rectangle shapes are easy to score, the scoring of the cube can be challenging.

#### Visuo-construction

Constructional praxis and praxis recall provide information about visuo-constructional ability and spatial memory that is useful in discriminating later stages of dementia. It is also useful in providing a non-verbal measure of memory [86]. Praxis/recall is assessed in some of HRS/HCAP studies using the CERAD battery [32, 33] copy of 4 shapes (circle, diamond, overlapping rectangles and cube). Constructional praxis is also assessed in the MMSE and MoCA. The CERAD constructional praxis task demonstrated substantial inter-rater reliability [38] and is quick and easy to administer. Scoring for the more complex figures (e.g. cube) can be sometimes be challenging however. Literacy is not a requirement, and visuo-construction tests are not limited by language constraints regarding cross-cultural applicability. Nonetheless, a recent review highlighted that tasks such as the construction praxis test and the clock drawing test from the MoCA are quite sensitive to differences in educational attainment and can be frustrating for some individuals who have received little or no formal schooling [87]. Fine motor impairments may also affect the validity of test results.

#### Proposed battery of cognitive tests for population and cross-country use

Drawing upon the strengths and limitations of the tests included in HRS/HCAP (see Table 3 for an overview), we indicate a comprehensive multidomain battery of cognitive tests for the assessment of cognitive capacity in the general population worldwide (Table 4). Our battery covers four essential cognitive domains including orientation, verbal memory, attention/executive function/working memory and language/fluency. The battery was selected with the caveat that it should be comprehensive and reliable while cost- and time-effective, implementable in low- and middle-income countries, inclusive of populations with low level of education, sensory and fine motor impairments to reduce the risk of sampling biases, culture-fair and deployable for cross-country comparisons. A test was chosen among other tests measuring the same cognitive domain if it offered the best ratio between its strengths and limitations. We further highlight five tests (in bold, Table 4) that would be appropriate for inclusion in a brief battery (11 minutes), which are sensitive to variations in cognitive capacity and include non-verbal in addition to verbal measures.

**Table 4:** Proposed battery of cognitive tests (in order of administration) for the assessment of cognitive capacity in the general population worldwide. The tests highlighted in bold represent the tests to be included in a shorter battery (11 minutes as opposed to 26 minutes)

Cognitive tests	Cognitive domain measured	Duration (minutes)^a^
1. Orientation (date, day, season, person, place)	Orientation	1.5
2. Object naming from description	Language/fluency	0.5
3. **HRS 10-word recall – Immediate (two trials)**	**Verbal memory**	**3**
4. **Animal naming**	**Language/fluency**	**1.5**
5. Count backwards from 20	Attention/executive function/working memory	0.5
6. Serial 7 s	Attention/executive function/working memory	1
7. **Symbol Digit Substitution (SDMT)**	**Attention/executive function/working memory**	**5.9**
8. **HRS 10-word recall – Delayed**	**Verbal memory**	**0.8**
9. Digit span	Attention/executive function/working memory	3
10. Logical memory – Immediate	Verbal memory	1
11. Follow instructions	Language/fluency	0.5
12. Raven’s matrices	Attention/executive function/working memory	6.1
13. Logical memory – Delayed	Verbal memory	0.5
Total duration		25.8

^a^The time in minutes is indicative and based on experience. It may vary depending on the cognitive capacity of the respondent.

By combining the scores obtained for each of the tests included in either battery, intrinsic cognitive capacity could be measured at an individual level, and the operationalisation of such a battery at a population level worldwide could also allow for development of population-based norms, which could serve in the future as reference values for use in community or primary care settings. The approach taken herein could assist the measurement of cognitive trajectories and the assessment of risk of accelerated cognitive decline at a population level, providing an ecologically valid alternative to more in-depth assessment of cognitive function aimed at diagnosing mild cognitive impairment and dementia.

### Limitations

The cognitive tests selected in this narrative review were originally thoroughly reviewed by expert advisory panel during the design of the HRS studies and shown to be feasible, reliable and valid for use at the population level [5, 88]. We acknowledge however that these tests are only a subset of all the cognitive tests appropriate for use in older adults. This narrative review aimed to shed light on important considerations to take into account when measuring cognitive capacity in older adult populations; however, systematic reviews defining the concept of cognitive capacity and comparing the psychometric properties of all the cognitive tests currently available to measure this concept are warranted.

## Conclusions

In this narrative review, we highlighted pertinent information about common cognitive testing instruments to help make informed decisions on cognitive capacity measurement. Drawing upon their strengths and limitations, we have indicated a comprehensive test battery to assess cognitive capacity, with the overarching goal of facilitating standardised operationalisation worldwide. Such standardisation is valuable for both research and policy making, affording the opportunity to evaluate the impact of biological, environmental, cultural, structural and political differences between countries and populations on cognitive capacity in later life. A brief test battery has also been suggested, with a view to inclusion in shorter surveys. This may be particularly useful when the primary goal is not to comprehensively evaluate cognitive capacity but instead to investigate the role of cognitive capacity in other fields of research (e.g. retirement/financial planning, and behavioural economics). Lessons learnt from conducting a nationally representative study of ageing and an HCAP study can inform the selection of tests for cross-country comparison with consideration to populations with low education, sensory and motor impairments. Equipped with adequate measurement tools and reliable health data, countries can determine public health action plans, prioritise resources and develop evidence-based policies to support optimal cognitive functioning in later life.

## Supplementary Material

aa-23-0376-File002_afad122
