# Supplementary material for: Assessing cognitive function in longitudinal studies of ageing worldwide: some practical considerations
Source: Age Ageing. 2023 Oct 30;52(Suppl 4):iv13–25. doi: 10.1093/ageing/afad122 (PMC10615066; doi:10.1093/ageing/afad122)
Supplement: aa-23-0376-File002_afad122 [file aa-23-0376-file002_afad122.docx]

World Health Organisation *Measurements of Healthy Ageing*

**Assessing cognitive function in longitudinal studies of ageing worldwide: some practical considerations.**

**SUPPLEMENTARY MATERIAL**

- **Appendix 1.** Description of the population-based longitudinal studies of aging included in this narrative review.
- **Appendix 2**. Detailed information per cognitive test used in the HRS and HCAP studies on accessibility, costs, equipment, duration, ease of administration, validity, reliability, literacy and numeracy requirements, adaptability to sensory impairments or disabilities, and cross-cultural and linguistic portability.
- **Appendix 3:** Supplementary References.

**Appendix 1.** Description of the population-based longitudinal studies of aging included in this narrative review.

| **Study description** | **HCAP sub-studies** |
| --- | --- |
| **10/66 studies (10/66)^1^** |  |
| The 10/66 is a population cohort which includes adults aged 65 years and over, living in 11 geographically defined urban and rural catchment area sites in eight low- and middle-income countries, across Latin American, China and India. Baseline data was collected between 2004 and 2006 for all centres (2007 - 2010 for Puerto Rico) and comprised 15,901 participants at risk of developing dementia. The follow-up period ranged between 3 to 5 years by site, with a total of 53,872 person-years of observation. A cross-culturally validated assessment was used across centers to collect information on participants’ demographics, health, lifestyle behaviors and biological samples. |  |
| **The Aging, Demographics and Memory Study (ADAMS)**^2^ |  |
| ADAMS is a supplement to the Health and Retirement Study, a representative national sample of the US older population, with the specific aim of conducting a population-based study of dementia. At baseline (2000-2002), a group of 1,770 HRS respondents, age 70 or older, was selected with a response rate of 56% (N= 856); Follow-up assessments (2002-2005, 2006-2008 and 2008-2009) included 252, 315 and 217 subjects respectively. In-person clinical assessments for dementia are conducted in the participant’s residence to collect information about their cognitive status and establish a research diagnosis of dementia, cognitive impairment with no demented or no cognitive impairment. Informant interviews also include detailed information about the participant’s cognitive and functional changes, medical and psychiatric history, current medication use, and current behavioral and psychiatric symptoms. Blood pressure and heart rate measures are also collected. |  |
| **The China Health and Retirement Longitudinal Study (CHARLS)**^3^ | CHARLS-HCAP^4^ |
| CHARLS is a nationally representative cohort of persons in China aged 45 or older and their spouses. The baseline survey was conducted in 2011-2012 and includes more than 17,500 individuals. The sample is followed every two years, using a face-to-face computer-assisted personal interview (CAPI), and provide information on demographic background, family, health status and functioning, health care and insurance, work and retirement and income. Data collection also includes objective physical measurements at every 2-year follow-up, and blood sample once in every two follow-up period. | The CHARLS-HCAP study was carried out at Wave 4 of CHARLS with a sample of 11,021 participants aged 60 and over. It is closely aligned with the HRS-HCAP study, comprising objective cognitive assessments and informant interviews. |
| **The Costa Rican Longevity and Healthy Aging Study (CRELES)**^5^ |  |
| CRELES is composed of two nationally representative longitudinal surveys of older adults in Costa Rica. It comprises five waves of data. The original CRELES Pre-1945 cohort includes more than 2,800 Costa Rica residents born in 1945 or before. Data collections were conducted in 2005, 2007, and 2009. The CRELES 1945–1955 Retirement Cohort (RC) includes about 4,200 Costa Rica residents born in 1945–1955 and their spouses. Interviews were carried out in 2011 and 2013. CRELES data contains information on self-reported physical health, psychological and cognitive health, living conditions, health behaviors, health care utilization, social support, work, and socioeconomic status. Objective physical measurements include anthropometrics and mobility. Urine and blood sample were collected during the 2005 and 2007 waves of the CRELES Pre-1945 and the 2010 wave of CRELES-RC. DNA is available for both cohorts. |  |
| **The English Longitudinal Study of Ageing (ELSA)**^6^ | ELSA-HCAP^7^ |
| ELSA is a panel study of a representative cohort of more than 18,000 persons aged 50 years and over in England. The study started in 2002 and the sample has been followed up every two years. Data is collected using computer-assisted personal interviews (CAPI) and self-completion questionnaires (SCQ), with additional nurse visits for objective physical measurements every four years. Topics include health and disability, economic characteristics, social networks and participation, household and family structure, biological markers and links to administrative data. | The study involved 1,274 ELSA participants aged 65 and older recruited from the parent ELSA sample. It was designed to leverage the HRS international network of studies to support joint epidemiological analysis of the etiology and impact of cognitive decline, and to make comparable national estimates of the dementia prevalence. Individuals with low cognition were oversampled. Fieldwork took place in 2017-2018. |
| **The Brazilian Longitudinal Study of Health, Ageing and Well Being (ELSI)**^8^ |  |
| ELSI is a longitudinal nationally representative study of about 10,000 people aged 50 years or older, residing in 5 Brazilian regions. The baseline survey was conducted between 2015 and 2016. Follow-up waves are planned every three years. Household, individual interviews and physical tests were administered at the respondents’ homes. ELSI data includes information on demographics, neighborhood, discrimination, work and retirement, family transfers, health behaviors, cognitive and mental health, use of medications and of health services. Blood samples are available for a subsample of the study participants. |  |
| **The Health and Aging in Africa: A Longitudinal Study of an INDEPTH Community in South Africa (HAALSI)**^9^ | HAALSI Dementia Study^10^ |
| HAALSI is a population-based study of adults aged 40 and over living in rural South Africa, with longitudinal follow-up at 3-year intervals. At baseline (2014-2015), the cohort consisted of 5,059 people. HAALSI collects information on cognitive and physical functioning, social networks, cardiometabolic disease and risk factors, HIV and HIV risk, and economic well-being. The study also includes anthropometric measures and blood biomarkers. The second wave was completed in 2019 and the third wave in 2021. | The study started in 2019 to investigate the epidemiology of dementia in South Africa. The sample includes 635 adults aged 50 years and older. The study battery overlaps with the HCAP and HRS studies. |
| **Healthy Ageing in Scotland (HAGIS)**^11^ |  |
| Healthy AGeing in Scotland (HAGIS) is a pilot study of people aged 50+ in Scotland. The HAGIS survey is largely based on the ELSA and NICOLA questionnaires. It collects data on cognitive health, financial literacy, personality and standard of living. The pilot study will recruit 1,000 participants aged 50 years or over from across mainland Scotland. |  |
| Health, Aging, and Retirement in Thailand (HART)^12^ |  |
| The HART study is biannual household survey of aging in Thailand. It includes 5,600 representative households from 5 regions and Bangkok and vicinity. Face to face interviews gather information on the health (e.g. physical, cognitive), employment, socioeconomic status, and social and financial supports within family networks of individuals aged 45 years and older. Each survey wave is conducted in every 2 years. The pilot baseline survey was conducted in 2009, the second pilot in 2011. Main waves were conducted in 2014, 2016 and 2019. |  |
| **The University of Michigan Health and Retirement Study (HRS)**^13^ | HRS-HCAP^14^ |
| HRS is a longitudinal panel study that has surveyed a representative sample of more than 30,000 Americans over the age of 50 and their spouses or partners since 1992. Biennial waves collect information on the changing health and economic circumstances associated with ageing, with a focus on income and wealth, physical, cognitive and mental health, use of healthcare services, work and retirement and family connections. Since 2006, data collection has expanded to include blood biomarkers and further measures on psychological health and social context. | The HCAP is a sub-study within the ongoing HRS which aims to measure and understand dementia risk by collecting a set of established cognitive and neuropsychological assessments and informant reports to better characterize cognitive function among older people. The baseline sample comprised 5,500 adults aged 65 and older and assessments started in 2016. |
| **Indonesia Family Life Survey (IFLS)^15^** |  |
| IFLS is a longitudinal survey in Indonesia, consisting of over 30,000 individuals living in 13 of the 27 provinces in the country. Baseline data was conducted in 1993–1994, the second wave in 1997–1998, the third wave in 2000, the fourth wave in 2007-2008 and the fifth wave in 2014-2015. The survey gathers information on individual respondents, their families, their households, the communities in which they live, their health (e.g. cognitive health, self-reported diseases) and education facilities they use. |  |
| **The Japanese Study of Aging and Retirement (JSTAR)**^16^ |  |
| JSTAR is a panel survey of around 4,200 people aged 50-75 from five cities/wards across Japan. It was initiated in 2005 and conducted every two years for four waves. The data was collected via face-to-face interviews using computers (CAPI). Additional information was also collected through a self-completed questionnaire (SCQ). The data contains information on the economic, social, and health conditions (including cognitive function) of the respondents. |  |
| **The Korean Longitudinal Study of Ageing (KLOSA)**^17^ |  |
| KLOSA is a prospective population-based study of more than 10,000 adults aged 45 years or older in the Republic of Korea (South Korea). The study was initiated in 2006 and participants have been surveyed biennially since then. The data is collected using computer-assisted personal interviews (CAPI) and self-completion questionnaire (SCQ) and includes information on family, health (e.g. diseases, cognition), employment, income, wealth, subjective expectations, including subjective life expectancy. |  |
| **The Longitudinal Aging Study in India (LASI)**^18^ | LASI-DAD^19^ |
| LASI is a nationally-representative, longitudinal survey which examines the health, economic, and social well-being of about 50,000 adults aged 45+ in India. The LASI pilot was launched in 2010. The first wave of data was collected in 2016. LASI consists of a household survey and an individual survey, using **computer-assisted personal interview (CAPI)** technique, which assess physical environment, household finances, demographics, family, social activities, health, health behaviors, work and pensions. The study also has objective physical measurements and collects blood samples. | The LASI Diagnostic Assessment of Dementia (LASI-DAD) aims to understand the epidemiology of cognitive impairment and dementia and societal impact. The sample comprises 4,000 adults aged 60 and older from the LASI cohort. Individuals at high risk of cognitive impairment were oversampled. |
| **Malaysia Ageing and Retirement Survey (MARS)**^20^ |  |
| MARS is a longitudinal study of 5,613 adults aged 40 and above in Malaysia. It was designed to ensure comparability with HRS, JSTAR, and SHARE. Baseline data (2018-2019) gathered information via computer-assisted personal interviews (CAPI) on the respondents’ family support and living arrangement, health (health status, healthcare utilization, psycho-social and cognition), work and employment, income and consumption, and housing and assets. Physical measurements were also collected and included height, weight, waist and hip circumference, blood pressure and grip strength. |  |
| **The Mexican Health and Aging Study (MHAS)**^21^ | Mex-Cog^22^ |
| MHAS is a longitudinal study of more than 15,000 adults 50 years and older in Mexico. The baseline survey was conducted in 2001, with follow-up interviews in 2003, 2012, 2015, and 2018. Data was collected through paper and pencil interviews and covers information on socioeconomic characteristics, migration, cognitive, mental and physical health, disability, use of healthcare services, housing characteristics and family networks. Anthropometric measures are also available for a subsample of respondents. | The Cognitive Aging Ancillary Study in Mexico (Mex-COG) began in 2016, using MHAS Wave 4 as the sampling frame. Mex-Cog assessments were carried out on 2,265 participants aged 55 years and older. Informant interviews were also gathered on a subset. Mex-Cog aims to gather vital information about cognitive aging in Mexico and the assessments overlap with the other HCAP studies. |
| **The Northern Ireland Cohort for the Longitudinal Study of Ageing (NICOLA)**^23^ | NICOLA-HCAP |
| NICOLA is an ongoing longitudinal cohort study of ageing in a sample of the Northern Ireland population aged 50 years or older. Data collection began in 2014 with a baseline sample of 8,500 adults. The NICOLA assessment is comprised of three elements: every second year, a computer-assisted personal interview (CAPI) is conducted at the participant’s home by a trained interviewer and a self-completion questionnaire (SCQ), which together capture information on health and social care utilization, health behaviors, medication, mental, physical and cognitive health, socioeconomic status and social circumstances, driving and travel. Every four years, participants take part in a health assessment where objective physical measurements such as their cardiovascular, cognitive and respiratory function as well as their physical and visual health are assessed. Biological samples with genetic analysis are also available. | The study began in 2022 and has a target sample of 1,000 adults aged 65 years and older recruited from the parent NICOLA study. The NICOLA-HCAP battery of cognitive assessments is harmonized with TILDA-HCAP, ELSA-HCAP and HRS-HCAP. Informant interviews are also carried out. |
| **Study on global AGEing and adult health (SAGE)**^24^ |  |
| The World Health Organization’s SAGE is a longitudinal study which collects information on the health (e.g. cognitive, physical) and well-being of over 40,000 adults aged 50+ in China, Ghana, India, Mexico, Russia and South Africa. It also includes a smaller comparative cohort of adults aged 18-49 years. The survey was established in 2004 and conducted 3 follow-up (2010, 2013 and 2015). |  |
| **The Ageing and Retirement in Europe (SHARE)**^25^ | SHARE-HCAP |
| SHARE is a multinational survey which includes data of more than 140,000 individuals aged 50 or older and their partners from 27 European countries. The survey was initiated in 2004/2005 and follow-up waves were conducted every second year since then. Data include information on self-reported physical, psychological, cognitive and behavioral health, socio-economic circumstances and family network. Objective physical measurements and blood biomarkers are also available for some countries and waves. | The SHARE-HCAP study aims to explore how differences in health and social circumstances across continental Europe impact cognitive health in later life. It will overlap in content with the other HCAP studies and will comprise 2,500 participants across 5 countries: Denmark, Czech Republic, Germany, France and Italy. Fieldwork is ongoing. |
| **The Irish Longitudinal Study on Ageing (TILDA)** ^26,27^ | TILDA-HCAP |
| TILDA is a prospective cohort study of community-dwelling adults aged 50 years and over and their spouses in Ireland. Baseline data from the 8,504 participants was collected between October 2009 and July 2011. Biennial data collection includes two components: a computer-assisted personal interview (CAPI) administered by trained social interviewers in the participants’ own homes and a self-completion questionnaire (SCQ) completed in the participants’ own time. A comprehensive health assessment delivered by trained research nurses in a dedicated health centre, or a modified version delivered in the participant’s home also take place every second wave which collects objective physical measurements. TILDA has rich data on neurocognitive function, mental and physical health, cardiovascular function, kidney function, locomotion, falls, fear of falling, vision, socioeconomic status and social circumstances obtained during multiple waves of data collection. | The TILDA-HCAP began in 2021 and is closely harmonized with the HRS-HCAP, ELSA-HCAP and NICOLA-HCAP. The study aims to achieve a final sample of 1,400 adults aged 65 and older who are part of the TILDA cohort. The study includes a detailed battery of cognitive assessments and informant interviews. |

**Appendix 2**. Detailed information per cognitive test used in the HRS and HCAP studies on accessibility, costs, equipment, duration, ease of administration, validity, reliability, literacy and numeracy requirements, adaptability to sensory impairments or disabilities, and cross-cultural and linguistic portability.

| **Cognitive domain** | **Cognitive tests** | **Accessibility** | **Costs** | **Equipment** | **Duration** | **Ease of administration** (Clarity of instructions, ease of scoring, personnel training and quality control) | **Validity, reliability:** construct validity, sensitivity/ specificity, test-retest, inter-rater and intra-rater reliability, practice effects | **Requirements** (Literacy, numeracy; adaptability to sensory/ physical impairments) | **Cross-cultural and linguistic portability** |  |  |
| --- | --- | --- | --- | --- | --- | --- | --- | --- | --- | --- | --- |
| Global cognitive function | MMSE | Online via the Psychological Assessment Resourcing website. | Kits $219 - $277 (manual, forms and guide) | Paper (forms) and pencil | 10-15 minutes | Clear instructions. User-friendly forms that are relatively easy to score. Serial 7s, World spelling and picture drawing may require quality control to ensure standardized scoring across administrators. Free training available on the training portal. The MMSE can be administered by anyone who has been trained to assess cognitive impairment and who is familiar with the administration instructions. | Adequate to excellent internal consistency and construct validity; adequate to excellent test-retest, although susceptible to learning effects^28-30^, and adequate to excellent inter-rater and intra-rater reliability in populations with Alzheimer’s disease and dementia^31-34^ and in community dwelling older adults.^35-38,32,39^ Pooled sensitivity and specificity for detecting dementia: 0.80-0.88 sensitivity and 0.81-0.89 specificity^40-42^ and 0.62-0.66 sensitivity and 0.63-0.87 specificity for detecting mild cognitive impairment^40,41,43^. | **Biased against people with low levels of education or with poor literacy and/or numeracy skills. To address this, the** Hindi version (H-MMSE) was developed by Ganguli et al.^44^. Serial 7s was replaced by a subtraction task in the form of a story; World spelling was replaced by listing forward and backward the days of the week. The reading task (Read this: “Close you eyes” and do what it says) was replaced by an oral instruction (“Now look at my face and do what I do”). Sentence writing was substituted by asking the respondent to say something about their house. The pentagon figure was replaced by a diamond in a square**.**  **Not appropriate for individuals with severe visual impairment.** To accommodate visual impairment, naming can be administered via touching. | The MMSE is translated in 75 languages. MMSE-2 (new version) is available in three Spanish versions, German, French, Dutch, Chinese, Russian, Italian and Hindi.  Biased towards conceptual and geographical organisation of Western countries. To address this, the H-MMSE includes modifications to Orientation to time and Orientation to place to adapt to the cultural milieu, specifically to rural areas. “Which year is it?” was replaced to “Is it morning, afternoon or evening?” and questions asking about home address, storeys, counties were replaced by questions about e.g. “district”, “post-office” and “village”. |  |  |
|  | MoCA (Montreal Cognitive Assessment) | Accessible online. Electronic and paper copies can be downloaded. Clinical and educational use without permission. Research and commercial/ pharma must get permission. | Free for academics and research use. | Pen and paper. There is also an electronic version available for download for tablets; a telephone version and an audiovisual version for videoconference use. | 10 minutes (a 5-minute ‘Mini MoCA’ available for telephone administration) | Relatively straightforward to administer. Brief online certification and training required for permission to be granted (free for students and academics). Pen and paper full MoCA involves some effort and attention to detail on the part of the administrator to calculate the scores. Eased in the electronic version where scores are automatically calculated. Cube drawing, Serial 7s and verbal fluency may require quality control to ensure standardized scoring across administrators. | Adequate to excellent internal consistency and construct validity; adequate to excellent test-retest, although susceptible to learning effects ^28,33,34^, and adequate to excellent inter-rater and intra-rater reliability in populations with Alzheimer’s disease and dementia^31-34^ and in community dwelling older adults^35-38,32,39^ 0.81-0.89 sensitivity and 0.73-0.75 specificity^40,43^ to screen for mild cognitive deficits. | Literacy is a consideration for the full MoCA. Subjects with lower education levels may have difficulty performing the trail making test. The authors have now developed a version for use in populations with illiteracy or low literacy (MoCA Basic).  **Certain items are not appropriate for individuals with sensory impairment.** | The MoCA is translated into 36 languages, a process which involved linguistic and cultural modifications. For example, substituting animals in the naming task to include animals more commonly seen or part of the general vocabulary (e.g. an owl instead of a rhinoceros). |  |  |
|  | Telephone Interview for Cognitive Status (TICS)  TICS-3 items | Accessible online via the Psychological Assessment Resourcing website. | $138 (manual and forms) | Pen and paper | 10 minutes; <2 minutes for the TICS 3 items | Relatively straightforward to administer. Specific instructions for administration and scoring criteria are given in the record forms. Serial 7s may require quality control to ensure standardized scoring across administrators. 3 questions only given verbally for the Brief CSI-D. | High level of internal consistency, test-retest and inter-rater reliability in older adults^45,46^, with vulnerability to learning effects^47^. 0.92 sensitivity and 0.66 specificity^48^ to detect dementia. | Designed to be administered via telephone, the TICS is particularly useful for examining individuals who are unable to read or write or visually impaired individuals. It may however present difficulties for hearing-impaired individuals if administered over the phone. | Translated into several languages including Finnish, French, German, Hebrew, Italian, Japanese and Spanish. Adaptations to the cultural and/or linguistic milieu to be considered (word list recall, orientation to place, person, naming). For example, “What do you call the kind of prickly plant that grows in the desert?” can be changed to “What does a king wear on his head?” when the word cactus is not well known in the country/ region of use. |  |  |
|  | Community Screening Instrument for Dementia (CSI-D)  Brief CSI-D 4 items | Accessible online (Stewart et al 2016 BMJOpen Appendix 1) | No costs | Pen and paper or laptop/ tablet if computerized | 15 minutes; < 2 minutes for the brief CSI-D 4 items | Relatively easy to administer. The animal naming task may present scoring challenges. 4 instructions only given verbally for the Brief CSI-D. | Achieves 87% sensitivity and 83% specificity for the detection of dementia^49-51^ across different socio-economic and cultural settings, with areas under the ROC curves ranging between 0.74 and 0.93. | No literacy requirement. | Culture-fair cognitive testing. Extensively validated across a variety of low and middle-income countries (e.g. Canada, USA, Jamaica, Asia, Latin America, the Caribbean, the Middle East, Africa, India, China, South East Asia). Translated into several languages including Cree, Urdu, Arabic, Chinese, Taiwanese, Swahili and Persian. Minor changes to questions required to ensure equivalent meaning across countries/languages. |  |  |
| Memory | Self-rated memory | Single harmonized question. | No costs | None | 30 seconds | Very quick and straightforward to administer and score. |  | No literacy requirement. | Easily translated into other languages. Cultural differences in the perceived stigmatization of dementia may influence responding and reduce cross-national comparability. |  |  |
|  | CERAD Word Recall - Immediate | Accessible by order from the website:  <https://sites.duke.edu/centerforaging/files/2017/04/CERAD-OrderForm.pdf> | CD-ROM: The CERAD Database and Assessment Instruments $600 | Flip book (available for purchase), pen | 5 minutes approximately | Straightforward to administer and score. | Good sensitivity and specificity for discriminating between normal cognition, mild impairment and dementia^52-55^, with high validity, interrater and test-retest reliability^52,56^. | No literacy requirement.  Standardized test administration can be difficult in individuals with hearing impairment. | CERAD neuropsychological battery translated in Arabic, Bulgarian, Chinese Cantonese and Mandarin, Dutch, English sign Language, Estonian, Finnish, French, German, Hindi, Italian, Japanese, Korean, Norwegian, Polish, Portuguese, Russian, Spanish. |  |  |
|  | CERAD Word Recall -Delayed |  |  |  | 1 minute |  |  |  |  |  |  |
|  | CERAD Word Recall & Recognition |  |  |  | 2 minutes |  |  | Requires literacy. |  |  |  |
|  | HRS 10-Word Recall - Immediate | Developed for HRS | No cost | No specialized materials | 1-5 minutes depending on the number of recall trials (this varies between studies from 1 to 3 trials) | Relatively straightforward to administer and score. | Immediate and delayed recall tasks loaded onto memory factor; provides evidence of the convergent and divergent validity of the HRS 10-word recall tasks^57^. | Does not require literacy.  Standardized tests administration can be difficult in individuals with hearing impairment. |  |  |  |
|  | HRS 10-Word Recall - Delayed |  |  |  | 1 minute |  |  |  |  |  |  |
|  | Logical Memory - Immediate | Available from  https://www.pearsonassessments.com/store/usassessments | Prices vary. As an example, kits available from $206.00  Pack of 25 response booklets is $61.20 | Paper forms (answer sheets), pen | 3 minutes | Can be challenging to accurately score in real-time. Accuracy of scoring is much improved by recording the tests administration and using the audio recording to correct scores after the assessment. | The Wechsler Logical Memory test exhibits good psychometric properties including test-retest reliability^58,59^, with the magnitude of practice effects varying as a function of age^60^. The East Boston Memory test shows reasonable sensitivity to dementia, correctly classifying 78% of outpatients in a memory clinic setting^61^. | Does not require literacy.  Standardized test administration can be difficult in individuals with hearing impairment. | Story context can be modified to use common names and streets and to reflect local context (e.g. as in LASI-DAD). |  |  |
|  | Logical Memory – Delayed |  |  |  |  |  |  |  |  |  |  |
|  | Logical Memory - recognition |  |  |  | 2 minutes | Individuals may be inclined to give a ‘don’t know’ response instead of a ‘yes’ or ‘no’ if unsure, which creates some challenges for consistent scoring |  |  |  |  |  |
|  | Prospective memory | Instructions are given in the HRS-family studies of ageing questionnaire available via the Gateway to Global Ageing (https://g2aging.org) | No licence required | Paper and pen/pencil | 1 minute (per set of instructions and test) | Prompt given only if the respondent cannot recall the task. Requires good training of administrators to standardise the delivery of the prompt in particular (e.g. waiting 5 seconds before prompting). | No information available. | Does not require literacy per se but PM1 does require that the individual can write their initials. | PM1 may not transfer to countries and cultures where naming conventions differ from Western Europe and US (e.g. Asia, Iceland, certain African countries). |  |  |
| Attention/ Working Memory/ Executive function | Letter cancellation | Materials available from  <https://strokengine.ca/en/assessments/single-letter-cancellation-test-slct/> | No licence required | Paper test materials, pencil/pen, timer | 5 minutes | Relatively easy to administer and score, though time and close attention must be paid when marking errors. | Good construct validity^72^. | Fine motor impairments may affect the validity of test results. Patients must be able to hold a pencil and recognize letters of the alphabet to complete. | Available in English, French and Hebrew, but not applicable to languages which use different writing systems (e.g. Hindi, Mandarin etc). |  |  |
|  | Symbol cancellation | Available from Lowery et al.^62^ | No license required | Paper test materials, pencil/pen, timer | 3 minutes | Easy to administer and relatively easy to score. Close attention needed when marking errors. |  | Fine motor impairments may affect the validity of test results. Patients must be able to hold a pencil.  No literacy requirement. | Appropriate for use in different cultural settings. However, cultural/educational variation in performance may exist^63^. |  |  |
|  | Symbol Digit Modalities Test (SDMT) | Available  from  https://www.parinc.com/Products/Pkey/395 | Cost varies depending on materials purchased  e.g. Kit (Manual 25 Autoscore Test Forms £169-171)  No other license required | Specific paper materials including carbon copy for scoring | 5 minutes maximum | Relatively easy to administer and score with correct materials. | Good construct validity, loading on processing speed factors with some degree of learning involved^73^, as well as good test-retest reliability^64^, though it may be influenced by practice effects if alternate test forms are not used^65^. | The test taker can give written or spoken responses, so SDMT can be used with individuals with fine motor impairments or speech disorders. | The SDMT is relatively culturally neutral – involving only geometric figures and numbers and can be administered to non-English speakers. Available with instructions in English, Dutch, Spanish. |  |  |
|  | Digit span | A short digit span forwards and backwards test is included as part of the MMSE.  A longer digit span is also included in some studies as a stand- alone task | See cost for MMSE.  No license required for stand-alone digit span test | No specific materials required | 1 minute for short digit span (MMSE)  2 minutes for longer digit span | Easy to administer and score. |  | Literacy is not a requirement.  Affected by numeracy and education level^76^.  Not appropriate for cases of severe hearing impairment. | Transferable to different language settings but not culture neutral. |  |  |
|  | Trail Making Test (TMT) | Available from  https://neuropsych.com/product/trail-making-test-adults/ | Free to use.  Relatively low cost materials  $75 for test manual and 100 tests | Specific paper forms, pencil/pen, stopwatch | 10 minutes | Quite challenging to administer and score. Administrator must monitor closely for trail deviations and quickly alert the examinee to the mistake. | Demonstrated adequate construct validity^66,67^ and test-retest reliability^68^, however it is susceptible to practice effects at short time intervals^69^, making the use of alternate forms optimal^70^. No practice effects were found over larger time intervals (e.g. 1 year)^71^. It also has excellent inter-rater reliability^72^. | Literacy and familiarity with English alphabet are required.  Not appropriate in cases of visual impairment. | Not culture neutral. |  |  |
|  | Color Trail test (1&2) | Available from https://www.parinc.com/Products/Pkey/77 | From $208 for introductory kit with test forms | Specific paper forms required, pencil/pen, stopwatch | 10 minutes | Quite challenging to administer and score. Administrator must monitor closely for trail deviations and quickly alert the examinee to the mistake.  Requires detailed training. | Demonstrated acceptable psychometric properties^73,74^. | Does not require literacy.  Instructions may be presented with visual cues only for individuals with hearing impairment.  Not appropriate in cases of severe visual impairment. | Designed to be a culturally neutral version of the TMT. |  |  |
|  | Three-stage command task | Part of the MMSE but may be administered as a stand-alone test | No licence required | Blank page required | Less than 1 minute | Easy to administer and score. |  | Slight variation in instruction where to put paper between studies and depending on physical ability (‘put it on your lap/on the table’). | Easily applicable and comparable across languages/cultures. |  |  |
|  | Raven’s Standard Progressive Matrices (17-item form) | Instructions available from Gateway to Global Ageing (https://g2aging.org)  Test available from https://www.pearsonassessments.com/store | Kits available from $262.50 | Raven’s test booklet | 6 minutes | Easy to administer and score. | Demonstrated adequate construct validity, loading on a general factor of intellectual functioning^75,76^, and adequate to excellent test-retest reliability^75,77-81^. Data on the psychometric properties of the 17-item version is lacking. | Literacy not a requirement. Appropriate for individuals with fine motor impairments and hearing impairments.  Not appropriate in cases of severe visual impairment. | Easily transferable to different countries and cultural settings. |  |  |
| Numeracy / Numeric ability | Serial 7 subtraction | Instructions are given in the HRS-family studies of ageing questionnaire available via the Gateway to Global Ageing (https://g2aging.org) | No costs | Pen and paper or laptop/ tablet to upload score if computerized | Less than one minute | Easy to administer. Scoring may be challenging at times requiring concentration and attention on the part of the administrator. For example, when errors are made, the administrator needs to re-assess correctness based on the last answer given which may be prone to errors. May require QC’ing to check that the scoring is correct. | Adequate construct and discriminant validity of the Serial 7s as measures of concentration and processing speed ^82^, heavily influenced by basic arithmetic skills^83^. | Numeracy task so may be challenging for individuals with low level of education.  Administered orally, therefore useful for examining individuals who are unable to read or write or individuals with sensory or fine motor impairments. | Ease of use for cross-country comparisons. |  |  |
|  | Backward counting | Instructions are given in the HRS-family studies of ageing questionnaire available via the Gateway to Global Ageing (https://g2aging.org) | No costs | Pen and paper or laptop/ tablet to upload score if computerized; a stopwatch if timed. | Less than one minute | Easy to administer. Scoring may be challenging at times requiring concentration and very close attention on the part of the administrator.  Backward counting can be particularly difficult to score when the respondent counts backward very fast from 100. May require quality control to check that the scoring is correct. |  | Numeracy task so may be challenging for individuals with low level of education.  Administered orally, therefore useful for examining individuals who are unable to read or write or individuals with sensory or fine motor impairments. | Ease of use for cross-country comparisons. |  |  |
|  | Number series | Instructions are given in the HRS-family studies of ageing questionnaire available via the Gateway to Global Ageing (https://g2aging.org) | No costs | Pen and paper given to the respondent; laptop/ tablet to upload score if computerized | Varies by number of questions asked and time taken to answer; approx <2 mins per question | Relatively easy to administer. The instructions may be difficult to understand, hence the importance of a practice run first to assess that the respondent understands the task. Easy to score. | Showed solid construct and discriminant validity for quantitative reasoning ability among community dwelling older adults^84^. | Numeracy task so may be challenging for individuals with low level of education. Note that the number series are automatically chosen from a list based on the correctness of the previous answers given by the respondent. The difficulty of the task is therefore adapted to the respondent’s numeracy skills.  Literacy requirement as the respondent is asked to write numbers on paper. | Ease of use for cross-country comparisons. | |  |
|  | Computation/ Logic | Instructions are given in the HRS-family studies of ageing questionnaire available via the Gateway to Global Ageing (https://g2aging.org) | No costs | Pen and paper or laptop/ tablet | Varies by number of questions asked and time taken to answer; approx < 2 mins per question | Easy to administer and score. |  | Numeracy task so may be challenging for individuals with low level of education.  Administered orally, therefore useful for examining individuals who are unable to read or write or individuals with sensory or fine motor impairments. | Ease of use for cross-country comparisons. | |  |
| Language/ fluency | Verbal fluency | Animal naming (semantic fluency) test Instructions are given in the HRS-family studies of ageing questionnaire available via the Gateway to Global Ageing (<https://g2aging.org>)  Phonemic fluency test is a component of the MMSE | No licence needed for animal naming  See cost for MMSE, | Pen, paper and timer needed for scoring | 1.5 mins | Very easy to administer. Can be a little more challenging to score if the respondent is very proficient at the task. The administrator must pay close attention for repetitions. | Valid measures of verbal ability and executive functioning^85^, however the contribution of different processes to task performance differs somewhat between semantic and phonemic fluency. Both types of fluency demonstrated adequate test-retest reliability^39,86^. | No literacy requirement though performance on the task is heavily influenced by educational attainment.  Not appropriate to use for respondents with speech difficulties. | Transfers well to other language settings without the need for modification.^21^ | | |
|  | Object naming | Three questions as part of the TICS. | No licence needed | No materials needed | 0.5 mins | Very easy to administer and score. |  | Literacy is not a requirement.  Can be administered to individuals with sensory and fine motor impairments. | Some cultural adaptation needed (‘cactus’ substituted for other item in certain countries, e.g. ‘coconut’ in LASI-DAD study). | | |
|  | Vocabulary: NART | Freely available  e.g.  [https://portal.dementiasplatform.uk](https://worldhealthorg-my.sharepoint.com/Users/joannefeeney/Documents/%0bhttps:/portal.dementiasplatform.uk %0d) | No cost | Word cards/word list required | 2 mins | Easy to administer, difficult to standardise scoring just to variations in accents. | The validity of the NART as a measure of prior (rather than current) intellectual functioning is well established^87,88^. | Not appropriate for use in populations where literacy levels are low.  Not suitable for visually impaired individuals.  Can cause discomfort for individuals with little-to-no formal education. | Not transferable to other cultural and language settings |  |  |
|  | Vocabulary: word definition | Adapted from WAIS for HRS – individuals must define 10 words  Adapted for HAIS from COGNITO computerized test battery – individuals define 5 words from multiple choice options | No cost | No specific materials | 0.5 to 1 min | Easy to administer and score. | Exhibits excellent psychometric properties^89^. | Not appropriate for use in where individuals have had little-to-no formal education. | Transferable to other language settings. |  |  |
|  | Word spelling | Component of the MMSE | No cost if used as stand-alone | No special materials needed | 0.5 mins | Easy to administer. Difficult to have a consistent way of scoring that is easily applicable to a large-scale study. | Backward word spelling showed good construct validity in reflecting information processing^82^. | Literacy is a requirement. | Transferable to different language settings. |  |  |
|  | Repeat sentences/ phrases | Part of the MMSE, but can be used as stand alone.  Two sentence repetitions are also included as part of MoCA. | See cost for MMSE/MoCA | No materials needed | 0.5 mins | Easy to administer and score.  Administrator must take care to enunciate clearly when delivering the sentence to be repeated. |  | Literacy is not a requirement.  Not appropriate for individuals with severe hearing impairment or speech difficulties. | Transferable to different cultural and language settings. |  |  |
|  | Write sentences | Component of the MMSE | See cost for MMSE | Pen and paper | 0.5 mins | Easy to administer and relatively easy to score. |  | Literacy is a requirement so not appropriate for populations where levels of literacy are low.  Fine motor impairment and visual impairment can also adversely affect ability to perform the task. | Transferable to different language settings but not culture neutral. |  |  |
|  | Reading comprehension (Read and follow instructions) | Component of the MMSE | See cost of MMSE | Instructions printed on page | 0.5 mins | Easy to administer, score and train. |  | Literacy is a requirement so not appropriate for populations where literacy levels are low.  Not suitable in cases of extreme visual impairment. | Transferable to different language settings but not culture neutral. |  |  |
| Orientation | Day, Month, Year, Day of the week | Instructions are given in the HRS-family studies of ageing questionnaire available via the Gateway to Global Ageing (https://g2aging.org) | No costs | Pen and paper or laptop/ tablet to upload score if computerized | < 2 mins | Easy to administer and score. |  | No literacy or numeracy requirements. Can be administered to individuals with sensory or fine motor impairments. | Questions about persons and places may need cultural/ linguistic adaptations. For example, the respondent may be asked about the prime minister and not about the president in countries where the prime minister has more power than the president; the respondent may not be asked about their house number in areas where street houses are not numbered and therefore alternative questions may need to be designed to assess similar concepts. |  |  |
|  | Season |  |  |  |  |  |  |  |  |  |  |
|  | Person |  |  |  |  |  |  |  |  |  |  |
|  | Place |  |  |  |  |  |  |  |  |  |  |
| Visuo-construction | Constructional praxis | 4 -items from the CERAD battery  Available to purchase https://sites.duke.edu/centerforaging/files/2018/10/CERAD-Order-Form-2018.pdf | CERAD manual and Assessment Instruments $600 | Paper copies of figures | 6 mins | Easy to administer. Can be difficult to score owing to some subjectivity in determining what constitutes an error. | The CERAD constructional praxis task demonstrated substantial inter-rater reliability^56^. | No literacy requirements. Not appropriate for individuals with extreme visual impairment. However, it is quite sensitive to differences in educational attainment and can be frustrating for some individuals who have received little or no formal schooling^90^.  Fine motor impairments may affect the validity of test results. | Culturally neutral – easily transferable to other countries/cultural contexts |  |  |
|  | Constructional praxis recall |  |  |  |  |  |  |  |  |  |  |

**Appendix 3:** Supplementary References:

1. Prina AM, Acosta, D., Acosta, I., Guerra, M., Huang, Y., Jotheeswaran, A. T., ... & Prince, M. Cohort profile: the 10/66 study. *International journal of epidemiology.* 2017;46(2):406-406i.

2. Langa KM, Plassman, B. L., Wallace, R. B., Herzog, A. R., Heeringa, S. G., Ofstedal, M. B., ... & Willis, R. J. The Aging, Demographics, and Memory Study: study design and methods. *Neuroepidemiology.* 2005;25(4):181-191.

3. Zhao Y, Yisong Hu, James P. Smith, John Strauss, and Gonghuan Yang. Cohort profile: the China health and retirement longitudinal study (CHARLS). *International journal of epidemiology.* 2014;43(1):61-68.

4. Zhao Y, Strauss, J., Chen X., Wang, Y. Gong, J., Meng, Q., Wang, G., Wang, H. *China Health and Retirement Longitudinal Study - Wave 4 User’s Guide.* National School of Development, Peking University; 2020.

5. Rosero Bixby L, William H. Dow, and Gilbert Brenes Camacho. Costa Rican Longevity and Healthy Aging Study. 2019.

6. Steptoe A, Elizabeth Breeze, James Banks, and James Nazroo. Cohort profile: the English longitudinal study of ageing. *International journal of epidemiology* 2013;42(6):1640-1648.

7. Cadar D, Abell, J., Matthews, F.E., Brayne, C., David Batty, G., Llewellyn, D.J. and Steptoe, A. Cohort profile update: the harmonised cognitive assessment protocol sub-study of the English longitudinal study of ageing (ELSA-HCAP). *International journal of epidemiology.* 2021;50(3):725-726i.

8. Lima-Costa MF, Fabíola Bof de Andrade, Paulo Roberto Borges de Souza, Anita Liberalesso Neri, Yeda Aparecida de Oliveira Duarte, Erico Castro-Costa, and Cesar de Oliveira. The Brazilian longitudinal study of aging (ELSI-BRAZIL): objectives and design. *American journal of epidemiology.* 2018;187(7):1345:1353.

9. Gómez-Olivé FX, Livia Montana, Ryan G. Wagner, Chodziwadziwa W. Kabudula, Julia K. Rohr, Kathleen Kahn, Till Bärnighausen et al. Cohort profile: health and ageing in Africa: a longitudinal study of an indepth community in South Africa (HAALSI). *International journal of epidemiology.* 2018;47(3):689-690.

10. Bassil DT, Farrell, M.T., Wagner, R.G., Brickman, A.M., Glymour, M.M., Langa, K.M., Manly, J.J., Salinas, J., Tipping, B., Tollman, S. and Berkman, L.F. Cohort Profile Update: Cognition and dementia in the Health and Aging in Africa Longitudinal Study of an INDEPTH community in South Africa (HAALSI dementia). *International Journal of Epidemiology.* 2022;51(4):e217-e226.

11. Douglas E, Alasdair Rutherford, and David Bell. Pilot study protocol to inform a future longitudinal study of ageing using linked administrative data: Healthy AGeing in Scotland (HAGIS). *BMJ open.* 2018;8(1):e018802.

12. Anantanasuwong D, Theerawanviwat, D., Siripanich, P. Panel Survey and Study on Health, Ageing, and Retirement in Thailand. 2019.

13. Sonnega A, Jessica D. Faul, Mary Beth Ofstedal, Kenneth M. Langa, John WR Phillips, and David R. Weir. Cohort profile: the health and retirement study (HRS). *International journal of epidemiology.* 2014;43(2):576-585.

14. Langa KM, Ryan, L.H., McCammon, R.J., Jones, R.N., Manly, J.J., Levine, D.A., Sonnega, A., Farron, M. and Weir, D.R. The health and retirement study harmonized cognitive assessment protocol project: study design and methods. *Neuroepidemiology.* 2020;54(1):64-74.

15. Frankenberg EaDT. The Indonesia Family Life Survey (IFLS): Study design and results from waves 1 and 2. 2000:47-64.

16. Ichimura H, Hideki Hashimoto, and Satoshi Shimizutani. Japanese study of aging and retirement. *RIETIDiscussion Paper Series* 2009.

17. Lee Y, Joung Hwan Back, Jinhee Kim, and Haewon Byeon. Multiple socioeconomic risks and cognitive impairment in older adults. *Dementia and geriatric cognitive disorders.* 2010;6:523-529.

18. Perianayagam A, Bloom, D., Lee, J., Parasuraman, S., Sekher, T.V., Mohanty, S.K., Chattopadhyay, A., Govil, D., Pedgaonkar, S., Gupta, S. and Agarwal, A. Cohort Profile: The Longitudinal Ageing Study in India (LASI). *International Journal of Epidemiology.* 2022.

19. Lee J, Khobragade, P.Y., Banerjee, J., Chien, S., Angrisani, M., Perianayagam, A., Bloom, D.E. and Dey, A.B. Design and methodology of the longitudinal aging study in India‐Diagnostic assessment of dementia (LASI‐DAD). *Journal of the American Geriatrics Society.* 2020;68:S5-S10.

20. Mansor N, Halimah Awang, N. F. A. Rashid, D. Gu, and M. Dupre. Malaysia ageing and retirement survey. *Encyclopedia of gerontology and population aging.* 2019:1-5.

21. Wong R, Michaels-Obregon, A. and Palloni, A. Cohort profile: the Mexican health and aging study (MHAS). *International journal of epidemiology.* 2017;46(2):e2.e2.

22. Mejia-Arango S, Nevarez, R., Michaels-Obregon, A., Trejo-Valdivia, B., Mendoza-Alvarado, L.R., Sosa-Ortiz, A.L., Martinez-Ruiz, A. and Wong, R. The Mexican Cognitive Aging Ancillary Study (Mex-Cog): study design and methods. *Archives of Gerontology and Geriatrics.* 2020;91:104210.

23. Neville C.E. CSM, Burns F. The Northern Ireland Cohort for the Longitudinal Study of Ageing (NICOLA). *Gu D, Dupre M (eds) Encyclopedia of Gerontology and Population Aging Springer, Cham.* 2019.

24. Chatterji S. World health organisation’s (who) study on global ageing and adult health (sage). *BMC proceedings.* 2013;7(4):1-1.

25. Börsch-Supan A, Martina Brandt, Christian Hunkler, Thorsten Kneip, Julie Korbmacher, Frederic Malter, Barbara Schaan, Stephanie Stuck, and Sabrina Zuber. Data resource profile: the Survey of Health, Ageing and Retirement in Europe (SHARE). *International journal of epidemiology.* 2013;42(4):992-1001.

26. Kearney PM, Hilary Cronin, Claire O'Regan, Yumiko Kamiya, George M. Savva, Brendan Whelan, and Rose Anne Kenny. Cohort profile: the Irish longitudinal study on ageing. *International journal of epidemiology.* 2011;40(4):877-884.

27. Donoghue OA, Christine A. McGarrigle, Margaret Foley, Andrew Fagan, James Meaney, and Rose Anne Kenny. Cohort profile update: The Irish longitudinal study on ageing (TILDA). *International journal of epidemiology* 2018;47(5):1398-13981.

28. Franco-Marina F, García-González, J.J., Wagner-Echeagaray, F., Gallo, J., Ugalde, O., Sánchez-García, S., Espinel-Bermúdez, C., Juárez-Cedillo, T., Rodríguez, M.Á.V. and García-Peña, C. The Mini-mental State Examination revisited: ceiling and floor effects after score adjustment for educational level in an aging Mexican population. *International psychogeriatrics.* 2010;22(1):72-81.

29. Galasko D, Abramson, I., Corey-Bloom, J. and Thal, L.J. Repeated exposure to the Mini‐Mental State Examination and the Information‐Memory‐Concentration Test results in a practice effect in Alzheimer's disease. *Neurology.* 1993;43(8):1559-1559.

30. Cooley SA, Heaps, J.M., Bolzenius, J.D., Salminen, L.E., Baker, L.M., Scott, S.E. and Paul, R.H. Longitudinal change in performance on the Montreal Cognitive Assessment in older adults. *The Clinical Neuropsychologist.* 2015;29(6):824-835.

31. Nasreddine ZS NAP, Valérie Bédirian, Simon Charbonneau, Victor Whitehead, Isabelle Collin, Jeffrey L. Cummings, and Howard Chertkow. The Montreal Cognitive Assessment, MoCA: a brief screening tool for mild cognitive impairment. *Journal of the American Geriatrics Society.* 2005;53:695-699.

32. Folstein MF FS, McHugh PR. Mini-mental state. A practical method for grading the cognitive state of patients for the clinician. *J Psychiatr Research.* 1975;12(3):189-198.

33. McDowell I, Kristjansson, B., Hill, G. B., Hebert, R. Community screening for dementia: The Mini Mental State Exam (MMSE) and modified Mini-Mental State Exam (3MS) compared. *Journal of Clinical Epidemiology.* 1997;50(4):377-383.

34. Tombaugh TN, McIntyre, N. J. The mini-mental state examination: A comprehensive review. *J Am Geriatr Soc.* 1992;40(9):922-935.

35. Molloy DWaS, T. I. M. Mental Status and Neuropsychological Assessment: A guide to the Standardized Mini-Mental State Examination. *International Psychogeriatrics.* 1997;9(1):87-94.

36. Feeney J, Savva, G.M., O’Regan, C., King-Kallimanis, B., Cronin, H. and Kenny, R.A. Measurement error, reliability, and minimum detectable change in the Mini-Mental State Examination, Montreal Cognitive Assessment, and Color Trails Test among community living middle-aged and older adults. *Journal of Alzheimer's Disease.* 2016;53(3):1107-1114.

37. Bruijnen CJ, Dijkstra, B.A., Walvoort, S.J., Budy, M.J., Beurmanjer, H., De Jong, C.A. and Kessels, R.P. Psychometric properties of the Montreal Cognitive Assessment (MoCA) in healthy participants aged 18–70. *International Journal of Psychiatry in Clinical Practice.* 2020;24(3):293-300.

38. Cumming TB, Lowe, D., Linden, T. and Bernhardt, J. The AVERT MoCA data: scoring reliability in a large multicenter trial. *Assessment.* 2020;27(5):976-981.

39. Tombaugh T. N. MNJ. The mini-mental state examination: a comprehensive review. *Journal of American Geriatric Society.* 1992: 922–935.

40. Tsoi KK, Chan, J.Y., Hirai, H.W., Wong, S.Y. and Kwok, T.C. Cognitive tests to detect dementia: a systematic review and meta-analysis. *JAMA internal medicine.* 2015;175(9):1450-1458.

41. Mitchell AJ. A meta-analysis of the accuracy of the mini-mental state examination in the detection of dementia and mild cognitive impairment. *Journal of psychiatric research.* 2009;43(4):411-431.

42. Lin JS, O’Connor, E., Rossom, R.C., Perdue, L.A. and Eckstrom, E. Screening for cognitive impairment in older adults: a systematic review for the US Preventive Services Task Force. *Annals of internal medicine.* 2013;159(9):601-612.

43. Breton A, Casey, D. and Arnaoutoglou, N.A. Cognitive tests for the detection of mild cognitive impairment (MCI), the prodromal stage of dementia: Meta‐analysis of diagnostic accuracy studies. *International journal of geriatric psychiatry.* 2019;34(2):233-242.

44. Ganguli M. R, G., Chandra, V., Sharma, S., Gilby, J., Pandav, R., Belle, S., Ryan, C., Baker, C., Seaberg, E., Dekosky, S. A Hindi version of the MMSE: the development of a cognitive screening instrument for a largely illiterate rural elderly population in India. *International Journal of Geriatric Psychiatry.* 1995;10:367-377.

45. Liu SI, Prince, M., Chiu, M.J., Chen, T.F., Sun, Y.W. and Yip, P.K. Validity and reliability of a Taiwan Chinese version of the community screening instrument for dementia. *The American journal of geriatric psychiatry.* 2005;13(7):581-588.

46. Desmond DW, Tatemichi, T.K. and Hanzawa, L. The Telephone Interview for Cognitive Status (TICS): reliability and validity in a stroke sample. *International journal of geriatric psychiatry.* 1994;9(10):803-807.

47. Ferrucci L, Del Lungo, I., Guralnik, J.M., Bandinelli, S., Benvenuti, E., Salani, B., Lamponi, M., Ubezio, C., Benvenuti, F. and Baroni, A. Is the telephone interview for cognitive status a valid alternative in persons who cannot be evaluated by the Mini Mental State Examination? *Aging Clinical and Experimental Research.* 1998;10(4):332-338.

48. Elliott E, Green, C., Llewellyn, D.J. and Quinn, T.J. Accuracy of telephone-based cognitive screening tests: systematic review and meta-analysis. *Current Alzheimer Research.* 2020;17(5):460-471.

49. Hall KS, Gao, S., Emsley, C.L., Ogunniyi, A.O., Morgan, O. and Hendrie, H.C. Community screening interview for dementia (CSI ‘D’); performance in five disparate study sites. *International journal of geriatric psychiatry.* 2000;15(6):521-531.

50. Prince M, Acosta, D., Chiu, H., Scazufca, M., Varghese, M. and 10/66 Dementia Research Group. Dementia diagnosis in developing countries: a cross-cultural validation study. *The Lancet.* 2003;361(9361):909-917.

51. Hall KS, Hendrie, H.C., Brittain, H.M., Norton, J.A., Rodgers, D.D., Prince, C.S., Pillay, N., Blue, A.W., Kaufert, J.N., Nath, A. and Shelton, P. The development of a dementia screening interview in 2 distinct languages. *International journal of methods in psychiatric research.* 1993;3(1):1-28.

52. Morris JC, Heyman A, Mohs RC, et al. The Consortium to Establish a Registry for Alzheimer's Disease (CERAD). Part I. Clinical and neuropsychological assessment of Alzheimer's disease. *Neurology.* 1989;39(9):1159-1165.

53. Fillenbaum GG, van Belle G, Morris JC, et al. Consortium to Establish a Registry for Alzheimer's Disease (CERAD): the first twenty years. *Alzheimers Dement.* 2008;4(2):96-109.

54. Barth S, Schonknecht P, Pantel J, Schroder J. [Mild cognitive impairment and Alzheimer's disease: an investigation of the CERAD-NP test battery]. *Fortschr Neurol Psychiatr.* 2005;73(10):568-576.

55. Sotaniemi M, Pulliainen V, Hokkanen L, et al. CERAD-neuropsychological battery in screening mild Alzheimer's disease. *Acta Neurol Scand.* 2012;125(1):16-23.

56. Welsh-Bohmer KAaM, R.C. Neuropsychological assessment of Alzheimer's disease. *Neurology.* 1997;49(3):S11-S13.

57. Blankson ANM, J. A Brief Report on the Factor Structure of the Cognitive Measures in the HRS/AHEAD Studies *Journal of aging research.* 2014.

58. Wechsler D, Holdnack JA, Drozdick LW. *Wechsler Memory Scale: Fourth Edition. Technical and Interpretive Manual.* San Antonio: Pearson; 2009.

59. Abikoff H, Alvir J, Hong G, et al. Logical memory subtest of the Wechsler Memory Scale: age and education norms and alternate-form reliability of two scoring systems. *J Clin Exp Neuropsychol.* 1987;9(4):435-448.

60. Lo AH, Humphreys, M., Byrne, G.J. and Pachana, N.A. Test–retest reliability and practice effects of the Wechsler Memory Scale‐III. *Journal of neuropsychology.* 2012;6(2):212-231.

61. Gfeller JDaH, G.J. The East Boston Memory Test: a clinical screening measure for memory impairment in the elderly. *Journal of clinical psychology.* 1996;52(2):191-196.

62. Lowery N, Ragland JD, Gur RC, Gur RE, Moberg PJ. Normative data for the symbol cancellation test in young healthy adults. *Appl Neuropsychol.* 2004;11(4):218-221.

63. Banerjee J, Jain U, Khobragade P, et al. Methodological considerations in designing and implementing the harmonized diagnostic assessment of dementia for longitudinal aging study in India (LASI-DAD). *Biodemography Soc Biol.* 2020;65(3):189-213.

64. Smith A. *Symbol digit modalities test.* 1973.

65. Hinton-Bayre A, Geffen G. Comparability, reliability, and practice effects on alternate forms of the Digit Symbol Substitution and Symbol Digit Modalities tests. *Psychol Assess.* 2005;17(2):237-241.

66. Reitan RM. Validity of the Trail Making Test as an indicator of Organic Brain Damage. *Perceptual and Motor Skills.* 1958;8(3):271-276.

67. Sanchez-Cubillo I, Perianez JA, Adrover-Roig D, et al. Construct validity of the Trail Making Test: role of task-switching, working memory, inhibition/interference control, and visuomotor abilities. *J Int Neuropsychol Soc.* 2009;15(3):438-450.

68. Bowie CRaH, P.D. Administration and interpretation of the Trail Making Test. *Nature protocols.* 2006;1(5):2277-2281.

69. Stuss DT, Stethem, L.L. & Poirier, C.A. Comparison of the three tests of attention and rapid information processing across six age groups. *Clin Neuropsychol.* 1987;1:139–152.

70. Wagner S, Helmreich I, Dahmen N, Lieb K, Tadic A. Reliability of three alternate forms of the trail making tests a and B. *Arch Clin Neuropsychol.* 2011;26(4):314-321.

71. Basso MR, Bornstein, R.A. & Lang, J.M. Practice effects on commonly used measures of executive function across twelve months. *Clin Neuropsychol.* 1999;13:283–292.

72. Spreen OS, E. A. Compendium of Neuropsychological Tests. *Administration, Norms, and Commentary edn Oxford University Press, New York.* 1998.

73. D'Elia LF, P. Satz, C. L. Uchiyama, and T. White. Colour Trails test. *Florida: Psychological Assessment Resources.* 1996.

74. Messinis L, Malegiannaki AC, Christodoulou T, Panagiotopoulos V, Papathanasopoulos P. Color Trails Test: normative data and criterion validity for the greek adult population. *Arch Clin Neuropsychol.* 2011;26(4):322-330.

75. Burke HR. Raven's Progressive Matrices: Validity, reliability, and norms. *The Journal of Psychology.* 1972;82(2):253-257.

76. Bingham WC, Burke, H.R. and Murray, S. Raven's Progressive Matrices: construct validity. *The Journal of Psychology.* 1966;66(2):205-209.

77. Queiroz-Garcia I, Amaral Espirito Santo H. Psychometric properties of the Raven’s Standard Progressive Matrices in a Portugese sample. *Portugese Journal of Behavioral and Social Research.* 2021;7(1):84-101.

78. Raven JC. *Mental tests used in genetic studies: The performances of related individuals in tests mainly educative and mainly reproductive.* Unpublished master’s thesis, University of London.; 1936.

79. Eysenck HJ. Types of personality: a factorial study of seven hundred neurotics. *Journal of Mental Science.* 1944;90(381):851-861.

80. Foulds GAR, J.C. Normal changes in the mental abilities of adults as age advances. *Journal of Mental Science.* 1948;XCIV(394):133-142.

81. Desai M. The test‐retest reliability of the Progressive Matrices Test. *British Journal of Medical Psychology.* 1952;25(1):48-53.

82. Williams MA, LaMarche, J.A., Alexander, R.W., Stanford, L.D., Fielstein, E.M. and Boll, T.J. Serial 7s and Alphabet Backwards as brief measures of information processing speed. *Archives of Clinical Neuropsychology.* 1996;11(8):651-659.

83. Karzmark P. Validity of the serial seven procedure. *International journal of geriatric psychiatry.* 2000;15(8):677-679.

84. Fisher GG, J. J. McArdle, R. J. McCammon, A. Sonnega, and D. Weir. New measures of fluid intelligence in the HRS. *Ann Arbor, Michegan: Institute for Social Research, University of Michigan.* 2013.

85. Shao Z, Janse E, Visser K, Meyer AS. What do verbal fluency tasks measure? Predictors of verbal fluency performance in older adults. *Front Psychol.* 2014;5:772.

86. Harrison JE, Buxton, P., Husain, M. and Wise, R. Short test of semantic and phonological fluency: Normal performance, validity and test‐retest reliability. *British Journal of Clinical Psychology.* 2000;39(2):181-191.

87. Dykiert DaD, I.J. Retrospective validation of WTAR and NART scores as estimators of prior cognitive ability using the Lothian Birth Cohort 1936. *Psychological assessment.* 2013;25(4):1361.

88. Crawford JR, Deary IJ, Starr J, Whalley LJ. The NART as an index of prior intellectual functioning: a retrospective validity study covering a 66-year interval. *Psychol Med.* 2001;31(3):451-458.

89. Iverson G, Sherman, E., Myers, B. and Adams, R. Clinical usefulness of the WAIS-R seven-subtest short form in persons with lateralized brain lesions. *Journal of Cognitive Rehabilitation.* 2000.

90. Franzen S, van den Berg, E., Goudsmit, M., Jurgens, C.K., Van De Wiel, L., Kalkisim, Y., Uysal-Bozkir, Ö., Ayhan, Y., Nielsen, T.R. and Papma, J.M. A systematic review of neuropsychological tests for the assessment of dementia in non-western, low-educated or illiterate populations. *Journal of the International Neuropsychological Society.* 2020;26(3):331-351.
